# Red Cell Distribution Width and Platelet Count as Biomarkers of Pulmonary Arterial Hypertension in Patients with Connective Tissue Disorders

**DOI:** 10.1155/2019/4981982

**Published:** 2019-06-02

**Authors:** Mattia Bellan, Ailia Giubertoni, Cristina Piccinino, Arnaldo Dimagli, Federico Grimoldi, Maurizio Sguazzotti, Michela Emma Burlone, Carlo Smirne, Daniele Sola, Paolo Marino, Mario Pirisi, Pier Paolo Sainaghi

**Affiliations:** ^1^Department of Translational Medicine, Università del Piemonte Orientale (UPO), Via Solaroli 17, 28100 Novara, Italy; ^2^Division of Internal Medicine, Immunorheumatology Unit, “Maggiore della Carità” Hospital, Corso Mazzini 18, 28100 Novara, Italy; ^3^Division of Internal Medicine, “Sant'Andrea Hospital”, Corso Abbiate 21, 13100 Vercelli, Italy; ^4^IRCAD (Interdisciplinary Research Center of Autoimmune Diseases), Novara, Italy; ^5^Division of Cardiology, “Maggiore della Carità” Hospital, Corso Mazzini 18, 28100 Novara, Italy

## Abstract

**Introduction/Objective:**

In the present paper, we aimed to test the value of the red cell distribution width (RDW) coefficient of variation as a candidate biomarker for pulmonary arterial hypertension (PAH) in patients with connective tissue disorders (CTD), correlating it with the degree of cardiopulmonary impairment in these patients.

**Methods:**

The study population included *N* = 141 patients with CTD and *N* = 59 patients affected by pulmonary hypertension of other etiologies, all referred to the Pulmonary Hypertension Clinic of the Cardiology Division of an Academic Hospital in Northern Italy for evaluation (including right catheterization). Clinical, instrumental, and laboratory data were collected and related to RDW and other full blood count indexes.

**Results:**

Twenty out of 141 CTD patients (14%) received a diagnosis of PAH. In comparison to those without PAH, CTD patients with PAH displayed a larger RDW (14.9% (13.5-17.2) vs. 13.8% (13.1-15.0); *p* = 0.02) and a lower platelet count (205 (177‐240) × 10^9^/l vs. 244 (197.5‐304.2) × 10^9^/l; *p* = 0.005). Moreover, with respect to CTD patients without PAH, RDW was significantly larger also in PH of other etiologies. In contrast, the platelet count was significantly lower only in CTD-related PAH, with a value > 276 × 10^9^/l being 100% sensitive in ruling out PAH. Finally, RDW, but not the platelet count, was related directly to systolic pulmonary arterial pressure (*r* = 0.381; *p* = 0.0008) and right ventricle diameter (*r* = 0.283; *p* = 0.015) and inversely to diffusing capacity of the lung for carbon monoxide (*r* = −0.325; *p* = 0.014).

**Conclusion:**

RDW is a promising candidate biomarker for the screening and the prognostic stratification of PAH in CTD patients.

## 1. Introduction

Pulmonary hypertension (PH) is a clinical entity that comprises multiple conditions of different etiologies and pathophysiology, defined by the presence of a mean pulmonary arterial pressure (mPAP) equal to or greater than 25 mmHg, measured during invasive right heart catheterization (RHC) at rest [1]. Although reliable estimates of prevalence are lacking, PH as defined above is not uncommon. Group 1 PH (i.e., pulmonary arterial hypertension (PAH), characterized by a pulmonary artery wedge pressure ≤ 15 mmHg), on the other hand, is a rare disease that may complicate connective tissue diseases (CTD) [2]. The estimated prevalence and incidence of PAH are around 15-60 cases and 5-10 cases per million adult population, respectively [3]; roughly, half of these cases occur in association with CTD, with a prevalence of 21-29% in mixed connective tissue disease (MCTD), up to 14% systemic lupus erythematosus (SLE) [4], and close to 20% in systemic sclerosis (SSc) [5]. Lower rates have been reported in Sjogren's syndrome (SS) [4] and in dermatomyositis/polymyositis (PM/DM) [6].

Consensus exists that these high-risk patients should be screened for PAH, since CTD-related PAH has a severe prognosis, even poorer than the idiopathic form [7]; in fact, PAH is the leading cause of death in systemic sclerosis [8]. Moreover, early treatment of PAH patients leads to improved outcomes, whereas treatment delay is associated with clinical worsening [9]. The screening strategy most used today is based on the application of a two-step algorithm (DETECT) on systemic sclerosis patients, which is 97% sensitive and 35% specific for the diagnosis of PAH [10]. Despite the relatively good performance of the DETECT algorithm, no universally agreed approach by which to screen these patients exists, and novel biomarkers of PAH are actively pursued.

The red cell distribution width (RDW) coefficient of variation, a measure of the variability in size of circulating erythrocytes, is routinely reported as a component of the complete blood count and has been used in the differential diagnosis of anemia [11]. Recently, RDW has been shown to stratify prognosis among patients with idiopathic and thromboembolic PAH [12, 13]. Moreover, RDW has been proposed as a predictor of PAH in SSc and in SS [14, 15].

In the present paper, we aimed to evaluate RDW as a candidate biomarker for PAH in CTD patients and to correlate it with the severity of cardiopulmonary impairment.

## 2. Methods

### 2.1. Patients

We performed a cross-sectional observational study. From October 1^st^, 2016, to April 20^th^, 2018, we recruited 141 consecutive patients, affected by CTD and referred to the Pulmonary Hypertension Outpatient Clinic of the Cardiology Department, A.O.U. “Maggiore della Carità,” Novara, Italy.

We applied the following exclusion criteria:

- Age < 18 years
- Refusal to participate in the study
- Impossibility to undergo the required cardiopulmonary assessment

We also recruited 59 patients affected by PH of other etiologies, applying the same exclusion criteria, to check whether the predictive factors identified in CTD-related PAH were valid in other clinical settings.

### 2.2. Main Outcome Variable

Following echocardiography estimation of systolic pulmonary artery pressure (sPAP), right heart catheterization (RHC) was performed to confirm PH, when appropriate. PAH was defined by mean pulmonary artery pressure (mPAP) ≥ 25 mmHg, pulmonary capillary wedge pressure ≤ 15 mmHg, and pulmonary vascular resistance > 3 Wood Units. Whenever contraindications to RHC occurred, PH was diagnosed based on echocardiography-estimated sPAP ≥ 35 mmHg and additional high-probability criteria, according to 2015 ESC/ESR guidelines. In the control group, PH etiology was carefully searched for, and patients were classified according to the Nice 2013 criteria [1].

### 2.3. Procedures

All patients underwent a comprehensive medical history and a thorough physical examination, aimed at assessing the presence and severity of signs and symptoms compatible with PH. Cardiovascular risk factors and other comorbidities were investigated. Past medical history and drug history were recorded. Rheumatologic disease history was recalled through an extensive review of the medical records.

Complete blood count (CBC), serum creatinine, C-reactive protein (CRP), and brain natriuretic peptide (BNP) were measured. The serum autoantibody profile was examined. Further assessment of these patients consisted of

- 12-lead electrocardiogram with 6 limb and 6 precordial leads with paper speed set at the standard rate of 25 mm/s
- six-minute walking distance (6MWD), which measures the distance that the patient could quickly walk over a total of six minutes on a hard, flat surface
- posteroanterior and lateral chest X-rays and, when necessary, high-resolution computed tomography (HRCT) (to rule out interstitial lung disease)
- pulmonary function tests (PFTs), performed on the same day the patient attended the PH clinic. The spirometry was performed using standardized equipment and technique with a spirometer, which measured the amount of air the subject exhaled and the rate at which it was exhaled. The device was connected to a computer employing “Medisoft ExpAir 1.28.20” to convert the signals into numerical values and graphics. The following standardized measurements were evaluated: forced vital capacity (FVC), forced expiratory volume in the 1st second (FEV1), FEV1/FVC (%) (also known as the Tiffeneau index)
- the diffusing capacity of the lung for carbon monoxide (DLCO), measured with the single-breath Jones-Meade protocol. Once the mouthpiece and the nose clip were in place, the subject made a maximal expiration and then, with a maximal inspiration, inhaled a gas blend containing carbon monoxide (0.3%) and other inert gases (0.3% CH_4_). Then, the patient was asked to hold his/her breath for about 10 seconds and exhale afterwards. During the expiration, alveolar air was analyzed: the ratio between carbon monoxide in inspired gas and carbon monoxide in exhaled air determined the diffusion of carbon monoxide. Predicted DLCO corrected for haemoglobin and alveolar volume was assessed
- transthoracic echocardiography, performed using the Vivid 7 or E9 cardiovascular ultrasound machine by GE Medical Systems (Horten, Norway) with a 1.7/3.4 MHz tissue harmonic transducer. All data were obtained in standardized patient positions, according to the guidelines of the American Echocardiography Society. The exam was performed by an echocardiography expert in PH. The following parameters were studied [16]: sPAP, right atrium area (RAA), right ventricle diameter (RVD), and left ventricular ejection fraction (LVEF). LVEF has been evaluated with the modified Simpson method; systolic pulmonary artery pressure (sPAP) was estimated from the maximal velocity of tricuspid regurgitation (TR) and RAP (right atrial pressure) using Bernoulli's equation (sPAP = 4TR^2^ + RAP). RAP was estimated with the respiratory motion and the size of the inferior vena cava (IVC) from the subcostal view. TR jet was graded according to recommendations for the quantification of native valvular regurgitation
- right ventricle systolic function was evaluated by estimating the Tricuspid Annular Plane Systolic Excursion (TAPSE)

The study protocol was approved by the institutional ethical committee and conducted in strict accordance with the principles of the Declaration of Helsinki. Informed consent was obtained from all individual participants included in the study.

### 2.4. Statistical Analysis

Anthropometric, clinical, and biochemical data were recorded in a database and analyzed by the statistical software package MedCalc v.18.10.2 (MedCalc Software, Broekstraat 52, 9030, Mariakerke, Belgium). The measures of centrality and dispersion of data chosen were medians and interquartile range (IQR). Continuous variables were compared between groups by the Mann–Whitney and Kruskal-Wallis (K-W) tests. Exact Fischer's test and Pearson's *χ*^2^ test were used, as appropriate, to explore the associations of categorical variables. To test the diagnostic performance of RDW and platelet count, receiver operating characteristic curves were built, with calculation of the respective areas under the curve (AUC). The level of significance chosen for all statistical tests was 0.05 (two-tailed).

## 3. Results

Among the 141 patients with CTD, the following diagnoses were recorded: SSc (*N* = 102, 72.3%), SLE (*N* = 3, 2.1%), SS (*N* = 1, 0.7%), PM/DM (*N* = 13, 9.3%), MCTD (*N* = 10, 7.1%), and undifferentiated CTD (UCTD; *N* = 12, 8.5%). Out of 141, *N* = 20 received a diagnosis of PAH (14%): in *N* = 10 patients, the diagnosis was based on RHC data.

**Figure 1:**
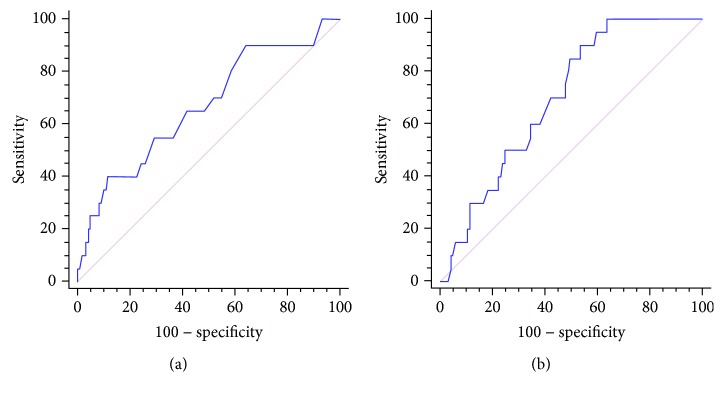
Receiver operating characteristic (ROC) curves for red cell distribution width (RDW) coefficient of variation and platelet count. In (a), we report the ROC curve of RDW for the diagnosis of PAH; in (b), we report the ROC curve of platelet count for the diagnosis of PAH.

In Table 1, we report the main features of these patients, as well as the comparison between patients with and without PAH. As shown in the table, the patients with PAH were older and characterized by a significantly higher BNP and sPAP; moreover, the creatinine clearance and the DLCO were significantly lower in the case of PAH. Interestingly, PAH was also associated with a larger RDW and a lower platelet count. We therefore analyzed the diagnostic accuracy of RDW and PLTs; in Figure 1, we report the respective ROC curves. An RDW value ≥ 16% was 40.0% sensitive and 88.3% specific for the diagnosis of PAH, with an AUC 0.666 (CI95%: 0.581-0.783; *p* = 0.015). A platelet count ≤ 276 × 10^9^/l was 100.0% sensitive and 36.3% specific for the diagnosis of PAH, with an AUC 0.697 (CI95%: 0.614-0.771; *p* = 0.0001). 5/20 patients were affected by chronic respiratory failure requiring continuous oxygen supplementation. This subset of patients had a trend, though not statistically significant, towards a larger RDW (17.7% (15.4-18.2) vs. 14.2% (13.4-16.0)) when compared to those with PAH and no chronic respiratory failure. Platelet count was not different between groups (data not shown).

**Figure 2:**
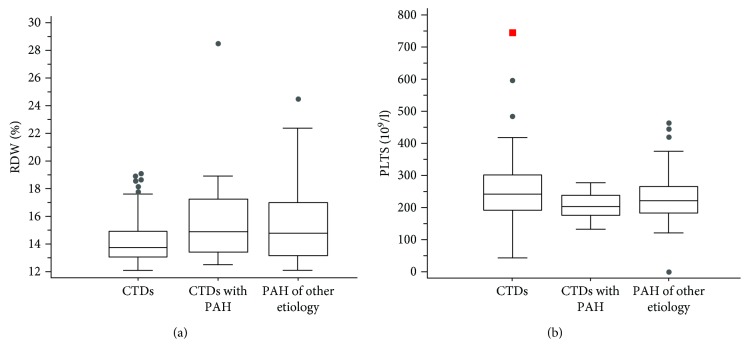
RDW and platelet values according to underlying diagnosis. The box and whiskers of RDW (a) and platelet (b) values according to the underlying diagnosis. RDW: red cell distribution width; PLTs: platelets; CTD: connective tissue diseases; PAH: pulmonary arterial hypertension.

We further tried to evaluate whether the increase in RDW and the decrease in platelet count were limited to CTD-related PAH or generalizable to other PH patients. In Table 2, we report the main features of the PH control group (Figure 2). With respect to CTD not complicated by PAH, RDW was significantly higher in both CTD-related PAH (14.9% (13.5-17.2)) and PH of other etiologies (14.8% (13.2-17.0); K-W 13.6; *p* < 0.001). On the contrary, the platelet count was significantly lower only in CTD-related PAH (205 (177‐240) × 10^9^/l; K-W 7.30; *p* = 0.02), being similar between PH of other etiologies (222 (184‐266) × 10^9^/l) and CTD not complicated by PAH (242 (194‐302) × 10^9^/l) (see also Figure 2).

Finally, we evaluated the correlations between RDW and platelet count measured in patients with PH and different clinical and instrumental parameters. In Table 3, we report the results of this analysis. Platelet count was not related to any of the functional and echocardiographic markers analyzed. On the contrary, RDW was inversely associated with DLCO and directly related to sPAP and right ventricle diameter.

## 4. Discussion

The results of the present study indicate that RDW, a simple parameter routinely obtained with the automated full blood count, may be of help in the diagnostic evaluation and prognostic stratification of CTD patients, with a specific focus on PAH. These data will be discussed at the light of the existing literature on the topic.

The rationale of the present study stems from previous reports, suggesting that red blood cells and platelets are possibly involved in the pathogenesis of several inflammatory diseases and may serve as diagnostic and prognostic biomarkers in this context [17]. Growing attention has been paid recently towards the prognostic implication of an increased RDW: in fact, this parameter has been used as an indicator of ineffective red cell production or haemolysis but has recently been identified as a predictor of poor prognosis in different cardiovascular and noncardiovascular diseases [18, 19]. With specific regard to PH, a high RDW predicts a poor prognosis in chronic thromboembolic PH and in PAH [12, 13] and may be a factor in CTD-related PAH, at least in Asian patients [14, 15]. Furthermore, in SSc patients, a higher RDW has been observed in those subjects with at least one clinical vascular involvement (acral ulcers, PAH, or renal crisis) [20]. Our data confirm and extend the abovementioned observations, suggesting that RDW may predict the presence of PAH in CTD patients; moreover, RDW has a potential prognostic implication, given its association with sPAP, right ventricular size, and DLCO, which are well-known prognostic markers in SSc-related PAH [21, 22]. We finally showed that RDW is elevated in PH belonging to classes different from class 1, therefore marking more generally PH rather than PAH in CTD. The reason why RDW is elevated under these circumstances is still unknown, although it might reflect the microvascular damage or the chronic inflammatory state, since elevation of RDW levels was shown to reflect the circulating levels of proinflammatory cytokines, such as tumor necrosis factor *α*, interleukin- (IL-) 1, and IL-6 [20].

We also reported a significantly lower platelet count in CTD patients with PAH; this observation might be explained by an intravascular thrombosis due to endothelial damage, leading to the local consumption of platelets [23]. However, a low platelet count seems to be more specifically associated with CTD-related PAH, since it was not observed in patients with other forms of PH and in those with uncomplicated CTD. Given that the platelet count was not associated with other important prognostic markers of PAH but has high sensitivity, its role may consist in the possibility of ruling out PAH, when above the threshold indicated.

It might be argued that the increased RDW and reduced platelet count belong to the development of hypoxia, leading to increased erythropoiesis at the expense of megakaryopoiesis. We have no data about blood gas analysis of our patients; however, those on oxygen supplementation because of chronic respiratory failure were not characterized by a different platelet count. However, a trend towards a larger RDW was observed. Hypoxia is, therefore, an intriguing potential explanation of these associations, deserving consideration in further studies.

A main limitation of the present study is related to the small sample size of our cohort which, being representative of a relatively rare complication of a rare disease, allowed us to enrol only 20 patients with CTD-related PAH. This significantly limits our possibility to draw definitive conclusions, particularly with regard to the identification of diagnostic thresholds. Therefore, the ROC curves presented have the value of an exploratory analysis, requiring confirmation in larger cohorts.

Finally, for the present study, the diagnosis of PAH was based on the 2015 ESC/ESR guidelines which defined pulmonary arterial hypertension in the presence of a mPAP ≥ 25 mmHg; this cut-off has been recently revised during the 2018 PH World Symposium. The new proposed definition of PAH is based on the detection of a mPAP ≥ 20 mmHg with a pulmonary vascular resistance ≥ 3 WU at RHC [24]. Our findings do not automatically extend to those patients with a mPAP 21-24 mmHg and, therefore, need to be confirmed using this new diagnostic threshold.

## 5. Conclusions

In conclusion, RDW is a promising tool for the screening and the prognostic stratification of PAH in CTD patients; its role in complementing and refining the approach by which we currently screen for PAH on these high-risk patients is worth to be established in future studies.

## Figures and Tables

**Table 1:** Main features of the study population and differences related to the presence of PAH. We report the main features of the whole study population; moreover, we compared CTD alone and CTD complicated by PAH. Categorical variables are shown as frequencies (%); continuous variables are shown as medians (IQR). Mann–Whitney and Fisher's exact tests were used as appropriate.

Variables	Entire CTD population (*N* = 141)	CTD without PAH (*N* = 121)	CTD with PAH (*N* = 20)	*p*
Age (years)	68 (57-76)	62 (52-72)	74 (69-79)	**0.0001**
Gender (F/M)	127 (90.1)/14 (9.9)	110 (90.9)/11 (9.1)	17 (85.0)/3 (15.0)	0.42
Hb (g/dl)	12.9 (11.8-14.0)	12.9 (11.7-13.8)	12.0 (11.6-13.8)	0.35
RDW (%)	14.0 (13.2-15.5)	13.8 (13.1-15.0)	14.9 (13.5-17.2)	**0.02**
PLTs (×10^9^/l)	228 (190-295)	244 (197.5-304.2)	205 (177-240)	**0.005**
BNP (pg/ml)	78.7 (35.3-182.1)	52.1 (29.7-102.2)	204.9 (81.0-465.5)	**<0.0001**
Creatinine clearance (ml/min)	82.5 (60.0-97.5)	89.0 (71.5-102.0)	65 (49.0-78.0)	**0.0005**
DLCO (ml/min/mmHg)	72.5 (52.0-87.0)	76.0 (64.0-87.2)	45.0 (40.2-54.7)	**<0.0001**
FEV1 (%)	96.0 (78.0-113.0)	101.0 (84.7-115.0)	103.0 (87.5-115.0)	0.98
FEV1/FVC (%)	107.0 (101.0-114.0)	109.0 (102.5-115.5)	107.0 (102.0-111.0)	0.41
sPAP (mmHg)	33 (25-45)	27 (23-31)	46 (42-57)	**<0.0001**
LVEF (%)	61.5 (57-66)	63.0 (59.0-67.0)	61.5 (55.5-65.5)	0.15

*N*: number; CTD: connective tissue diseases; PAH: pulmonary arterial hypertension; Hb: haemoglobin; RDW: red cell distribution width; PLTs: platelets; BNP: brain natriuretic peptide; DLCO: diffusion lung capacity for carbon monoxide; FEV1: forced expiratory volume in the 1st second; FVC: forced vital capacity; sPAP: systolic pulmonary artery pressure; LVEF: left ventricular ejection fraction.

**Table 2:** Main features of the PH control group. We report the main features of the control group with PH of other etiologies. Categorical variables are shown as frequencies (%); continuous variables are shown as medians (IQR).

Variables	*N* = 59
PH class (1/2/3/4/5)	17 (28.8)/10 (16.9)/7 (12.0)/10 (16.9)/15 (25.4)
Age (years)	73 (64-79)
Gender (F/M)	33 (56)/26 (44)
Hb (g/dl)	12.9 (11.9-14.8)
RDW (%)	14.8 (13.2-17.0)
PLTs (×10^9^/l)	222 (184-266)
BNP (pg/ml)	182.5 (77.7-299.5)
Creatinine clearance (ml/min)	70.0 (51.0-87.0)
DLCO (ml/min/mmHg)	53.0 (42.0-80.0)
FEV1 (%)	70.0 (57.0-89.5)
FEV1/FVC (%)	102.0 (93.2-111.0)
sPAP (mmHg)	53 (44-69)
LVEF (%)	60 (55-66)

*N*: number; class 1: pulmonary arterial hypertension; class 2: PH related to left heart disease; class 3: PH related to pulmonary disease; class 4: thromboembolic PH; class 5: others; Hb: haemoglobin; RDW: red cell distribution width; PLTs: platelets; BNP: brain natriuretic peptide; DLCO: diffusion lung capacity for carbon monoxide; FEV1: forced expiratory volume in the 1st second; FVC: forced vital capacity; sPAP: systolic pulmonary artery pressure; LVEF: left ventricular ejection fraction.

**Table 3:** Correlation between functional parameters and RDW and platelet count in patients with PAH.

Variable	RDW	Platelet count
6MWD	*r* = ‐0.051; *p* = 0.71	*r* = 0.021; *p* = 0.88
BNP	*r* = 0.045; *p* = 0.69	*r* = 0.006; *p* = 0.96
Creatinine clearance	*r* = ‐0.009; *p* = 0.94	*r* = 0.213; *p* = 0.06
Hb	**r** = ‐0.331;**p** = 0.002	*r* = ‐0.031; *p* = 0.79
sPAP	**r** = 0.381;**p** = 0.0008	*r* = 0.071; *p* = 0.54
LVEF	*r* = 0.089; *p* = 0.45	*r* = 0.182; *p* = 0.12
RAA	*r* = 0.199; *p* = 0.10	*r* = ‐0.081; *p* = 0.50
RVD	**r** = 0.283;**p** = 0.015	*r* = ‐0.038; *p* = 0.75
DLCO	**r** = ‐0.325;**p** = 0.014	*r* = 0.037; *p* = 0.79
FEV1	*r* = ‐0.179; *p* = 0.17	*r* = ‐0.169; *p* = 0.20
FEV1/FVC	*r* = ‐0.152; *p* = 0.25	*r* = ‐0.188; *p* = 0.15

RDW: red cell distribution width; 6MWD: 6-minute walking distance; BNP: brain natriuretic peptide; Hb: haemoglobin; sPAP: systolic pulmonary artery pressure; LVEF: left ventricular ejection fraction; RAA: right atrium area; RVD: right ventricle diameter; DLCO: diffusion lung capacity for carbon monoxide; FEV1: forced expiratory volume in the 1st second; FVC: forced vital capacity.

## Data Availability

The data used to support the findings of this study are available from the corresponding author upon request.
